# L'inhalation d’épingle à foulard: urgence pneumologique émergente

**DOI:** 10.11604/pamj.2015.22.277.6928

**Published:** 2015-11-23

**Authors:** Afafe Hebbazi, Wiam El Khattabi, Régis Bopaka, Hasna Jabri, Hicham Afif

**Affiliations:** 1 Service de Phtisiologie, Hôpital 20 Août, CHU Ibn Rochd, Casablanca, Maroc; 2 Service des Maladies Respiratoires, Hôpital 20 Août, CHU Ibn Rochd, Casablanca, Maroc

**Keywords:** Corps étranger, inhalation d´épingle à foulard, bronchoscopie, Foreign body, inhalation scarf pin, bronchoscopy

## Abstract

L'inhalation d’épingle à foulard (EF) est un phénomène de plus en plus fréquent dans les pays où les femmes portent le voile et dont les conséquences peuvent être graves. Le but de ce travail est de présenter notre expérience dans le diagnostic et la prise en charge de cette nouvelle entité clinique, de définir ses particularités et d'illustrer les dangers de la tenue d'une épingle à foulard dans la bouche. Soixante-dix cas d'inhalation d'EF ont été colligés en 8 ans (2007-2015). Il s'agit de 69 patientes, toutes voilées avec un âge moyen de 16,5 ans et d'un patient de 14 ans. Les patientes mettaient l’épingle entre leurs lèvres tout en fixant leurs foulards. L'inhalation a été accidentelle dans tous les cas. Le signe le plus fréquemment rapporté après inhalation était la toux. Le siège de l’épingle était plus fréquent au niveau de l'arbre bronchique gauche (52,9%). Une expulsion spontanée de l'EF a été notée dans 9 cas. La bronchoscopie souple, réalisée en première intention, dans 61 cas a permis l'extraction de l'EF, dans 83,6% des cas. Le recours à la bronchoscopie rigide a été nécessaire dans 4,9% des cas et à une thoracotomie également dans 4,9% des cas du fait d'une migration distale de l'EF. L'inhalation d’épingle à foulard représente une urgence pneumologique. Les cliniciens doivent être informés de cette forme distincte de corps étrangers intra-bronchiques, son diagnostic et les techniques de son extraction. L'extraction par bronchoscopie souple est une méthode efficace avec un taux de succès élevé. La prévention reste le meilleur traitement.

## Introduction

L'inhalation de corps étranger (CE) est un accident fréquent chez l'enfant, avec un pic de fréquence entre 1 et 3 ans et une prédominance masculine dans 2/3 des cas [1, 2]. Chez l'adulte, l'inhalation de CE est beaucoup plus rare que chez l'enfant, et se voit sur des terrains prédisposés (maladies neurologiques avec troubles de la déglutition ou du réflexe de toux, abus de sédatifs ou d'alcool) ou des situations propices (soins dentaires, quinte de toux, éclat de rire, sanglot…) [1, 3]. Elle constitue comme chez l'enfant, une urgence vitale avec une mortalité en phase pré-hospitalière de 3% environ [3]. Les CE les plus communément rencontrés sont de nature organique (cacahuètes, haricots secs…). Cependant, la nature des CE est influencée par les conditions socioculturelles et régionales. Dans le monde islamique, des épingles métalliques droites sont largement utilisées pour fixer le foulard autour de la tête chez les adolescentes et les jeunes femmes adultes. Celles-ci ont souvent la mauvaise habitude de tenir ces épingles dans la bouche pendant l'arrangement de leur voile. L'inhalation accidentelle de ces épingles aboutit le plus souvent à leur localisation dans l'arbre trachéo-bronchite chez ce groupe de patientes. L'extraction de ce type de corps étrangers pointus et potentiellement pénétrants est un défi qui nécessite une attention particulière. La mise au point de ce problème critique permettra sa prévention et sa prise en charge.

## Méthodes

Entre janvier 2007 et avril 2015, 70 cas d'inhalation d’épingle à foulard (EF) ont été colligés au service des maladies respiratoires de l'hôpital 20 Août de Casablanca. Cette étude rétrospective a concerné 69 jeunes filles portant, toutes, le voile islamique et a inclus un jeune garçon âgé de 14 ans ayant également inhalé une EF alors qu'il s'amusait en la portant dans sa bouche. La moyenne d’âge de nos patientes était de 16,5 ans avec des extrêmes de 10 à 27 ans. Aucun facteur favorisant: troubles de déglutition, maladie neurologique, neuromusculaire ou métabolique n'a été retrouvé. Par contre, une patiente souffrait d'une rétinopathie bilatérale avec cécité droite et une patiente venait d’être opérée pour un abcès hépatique dix jours avant son admission. Un asthme a été noté chez 4 patientes et une grossesse évolutive chez 2 autres. Toutes les patientes ont été soumises à un interrogatoire précis analysant l'ensemble des symptômes évocateurs d'inhalation de CE: toux, étouffement, raucité de voix, cyanose, hémoptysie, dyspnée et douleurs thoraciques. L'analyse de la cause, du temps écoulé depuis l'inhalation a été aussi obtenue. Toutes les patientes avaient un CE radio-opaque à la radiographie thoracique. La bronchoscopie souple, réalisée dans 61 cas, sous anesthésie locale avec une intubation orale, a constitué la procédure thérapeutique essentielle. Un contrôle radiologique a été systématiquement réalisé en post-endoscopie et la majorité des patientes ont pu quitter le service le jour même ou le jour suivant.

## Résultats

**Figure 1:**
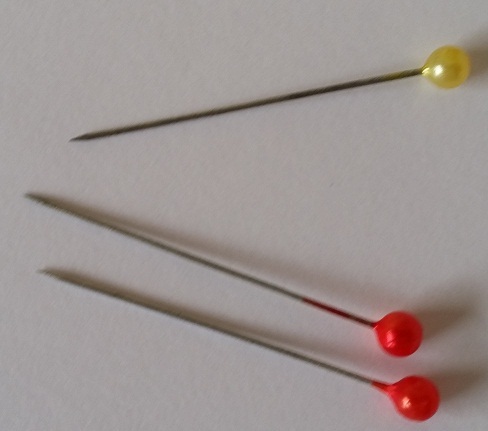
Epingles métalliques à bout capuchonné en plastique utilisées pour la fixation du foulard

**Figure 2:**
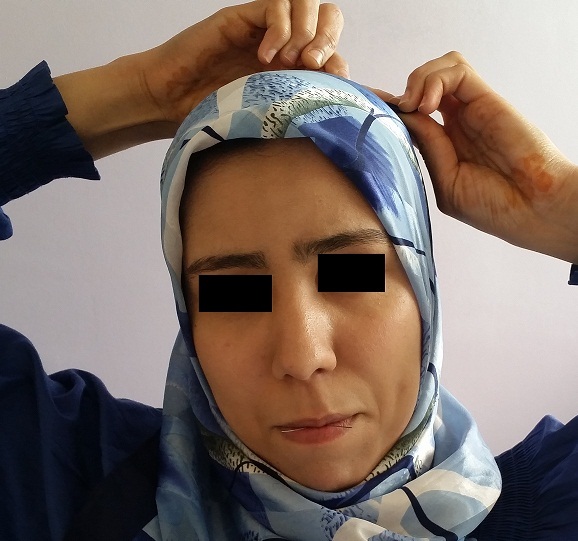
Position adoptée par les patients lors de la fixation du foulard

Le CE inhalé était une épingle à foulard (EF) dans tous les cas. Dans un cas, il s'agissait de l'inhalation simultanée de 2 épingles. L'EF comprend un corps métallique rectiligne, long de 3 à 4cm à bout métallique pointu d'un côté et bout capuchonné en plastique de l'autre (Figure 1). Les circonstances de survenue étaient la tenue d'une ou plusieurs épingles dans la bouche, entre les lèvres ou les dents au moment de la fixation du voile (Figure 2). L'inhalation d’épingle s'est faite accidentellement dans tous les cas, suite à une inspiration involontaire profonde secondaire à la parole, le rire, une toux ou un sentiment de surprise. Le délai moyen entre inhalation de l'EF et l'admission au service était de 4,5 jours avec des extrêmes de 2 heures à 6 mois. Les raisons du retard d'admission en hospitalier, noté dans certains cas, étaient relatives soit à une fausse impression d'avoir avalé le CE et donc l'attente de son expulsion par les voies naturelles, soit à une dissimulation de l'incident par la patiente de crainte d’être punie, soit à un niveau socio-économique et instructif bas avec une sous-estimation de la gravité de l'incident, ou encore à un éloignement d'une structure hospitalière. Après inhalation, le symptôme majoritairement rapporté était la toux dans le cadre du syndrome de pénétration, défini comme des quintes de toux explosives et soudaines. Une hémoptysie a été rapportée dans 16% des cas, un syndrome bronchique dans 13% des cas, une douleur thoracique dans 7,2% des cas et des nausées ou vomissements dans 2,8% des cas. Dans 14,5% des cas, les patientes étaient asymptomatiques. L'examen clinique a été normal dans tous les cas.

**Figure 3:**
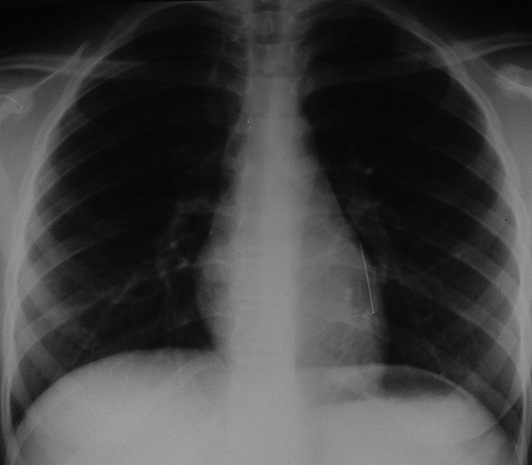
Radiographie thoracique de face: épingle à foulard se projetant sur la lobaire inférieure gauche

**Figure 4:**
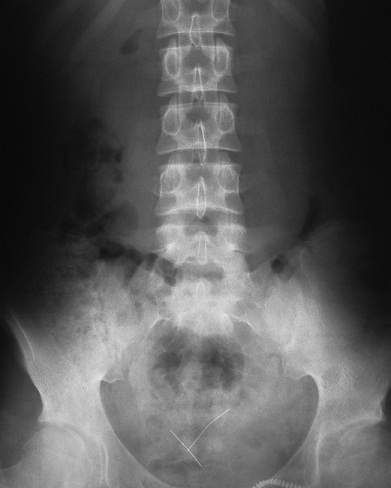
Abdomen sans préparation: inhalation simultanée de 2 épingles à foulard éliminées dans les voies digestives après un effort de toux

**Figure 5:**
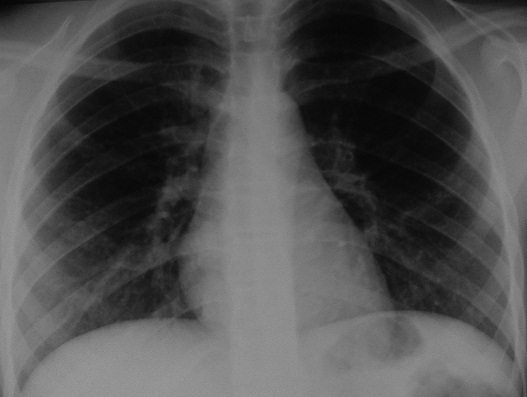
Radiographie thoracique après extraction de l’épingle à foulard

La radiographie thoracique face et profil a identifié le CE sous forme d'une opacité linéaire radioopaque, sans anomalie parenchymateuse ou pleurale associée, dans tous les cas (Figure 3). Elle a localisé le CE à gauche dans 37 cas (52,9%), à droite dans 32 cas (45,7%) et à projection trachéale dans 1 cas (1,4%). Un traitement médical comprenant une antibiothérapie (amoxicilline-acide clavulanique 3g/j), une corticothérapie de courte durée (prédnisolone) et une prémédication à base d'Atarax^®^ par voie orale a été prescrit dans la majorité des cas. Une expulsion spontanée du CE, par un effort de toux le déplaçant vers les voies pharyngées, entraînant ainsi sa déglutition puis son élimination quelques jours après dans les selles, a été notée dans 9 cas (12,8%) avant la réalisation d'une bronchoscopie souple. La bronchoscopie souple, réalisée en première intention dans 61 cas, a permis de visualiser l’épingle au niveau de la trachée dans 1 cas, la bronche principale gauche dans 12 cas, la lobaire inférieure gauche dans 22 cas, le culmen dans 1 cas, la bronche principale droite dans 2 cas, le tronc intermédiaire dans 3 cas et la lobaire inférieure droite dans 18 cas. Le CE n'a pas été visualisé dans 2 cas et un abdomen sans préparation a confirmé son passage vers les voies digestives (Figure 4). Des granulomes inflammatoires étaient présents dans 21 cas, des sécrétions mucopurulentes dans 16 cas et un saignement dans 3 cas. Dans tous les cas, l’épingle a été orientée tête perlée vers le bas et bout pointu vers le haut. Elle était incrustée dans la muqueuse par son extrémité pointue dans 22 cas (31,4%), incurvée dans un cas, friable et rouillée dans un autre cas. La manœuvre d'extraction par bronchoscopie souple, a été réussie en première intention dans 45 cas (73,8%), a été laborieuse dans 6 cas (9,8%) nécessitant le recours à une 2^ème^bronchoscopie souple dans 3 cas et à une 3^ème^ dans 3 autres cas. Au total, la bronchoscopie souple a permis l'extraction de l'EF dans 51 cas (83,6%). Elle était non fructueuse dans 10 cas (16,4%), nécessitant le recours à la bronchoscopie rigide dans 3 cas (4,9%) et à une thoracotomie dans 3 cas (4,9%) du fait d'une migration distale de l’épingle. Dans 2 cas (3,2%), l’épingle a été perdue, lors de son extraction, au niveau de la glotte, déglutie puis éliminée dans les selles 2 jours après. Elle n'a pas été visualisée lors de la bronchoscopie dans 2 autres cas (3,2%), localisée par un abdomen sans préparation au niveau intestinal. Un contrôle endoscopique après extraction n'a pas montré de réaction ou de lésion de la muqueuse bronchique. Le contrôle radiologique a montré une bonne évolution (Figure 5), à l'exception d'un cas de pneumothorax noté après une extraction laborieuse.

## Discussion

L'inhalation d'un CE est un accident fréquent chez les enfants dont les deux tiers sont des garçons, généralement plus turbulents, ayant tendance à découvrir les objets en les plaçant à la bouche, avec un pic de fréquence à 2 ans (CE végétaux) et un deuxième pic vers 6-8 ans (CE métalliques et plastiques) [4–6]. Chez l'adulte, les CE intrabronchiques sont rares, très souvent méconnus et de diagnostic souvent difficile. Un autre pic de fréquence est observé chez le sujet âgé de plus de 50 ans du fait de l'incidence des fausses routes [1, 7]. Chez l'adulte jeune et l'adolescent l'inhalation de CE est rare, elle complique le plus souvent soit un accident avec traumatisme facial (inhalation de fragments dentaires), soit des activités de bricolage au cours desquelles le sujet inhale accidentellement des objets (clous par exemple) qu'il tient entre ses dents [4]. Les CE communs incluent: aliments tels que les cacahuètes, les graines de pastèque ou du tournesol, billes, pièces de monnaie, prothèses dentaires et objets métalliques [8, 9].

Cependant, la nature du CE inhalé varie beaucoup en fonction des conditions socioculturelles et régionales, des habitudes alimentaires et éducatives des populations étudiées ainsi que du contexte religieux [2, 4, 8, 10]. En effet, au cours des 2 dernières décennies, un nouveau groupe distinct de CE intrabronchiques s'est, de plus en plus, individualisé dans les pays islamiques, en l'occurrence le Maroc. Le syndrome d'inhalation d'EF est une nouvelle entité clinique affectant les jeunes filles musulmanes portant le voile (écharpe, foulard traditionnel) [9, 11]. Des épingles droites sont largement utilisées pour fixer les foulards ou les écharpes autour de la tête et du cou. Il s'agit d’épingles métalliques longues de 3 à 4cm avec une tête en perle de plastique, radiotransparente, et une extrémité métallique pointue. Le port d'un voile et sa fixation correcte autour de la tête est une tâche assez complexe pour les jeunes filles. Celles-ci sont généralement moins attentives que les femmes adultes [7, 12]. En fait, pour garder les 2 mains libres tout en portant ou ajustant le foulard, il est habituel que la femme tienne une ou plusieurs épingles entre ses dents et les attache ensuite au foulard séquentiellement. La posture adoptée au cours de cette manœuvre, avec la tête inclinée vers l'arrière, tout en parlant, riant, toussant ou respirant profondément en même temps, favorise l'inhalation accidentelle de l’épingle [2, 4, 13–15]. Il s'agit d'une situation grave car cet objet pointu peut facilement migrer et s'enclaver en distalité rendant son extraction parfois difficile par endoscopie d'où l'indication, dans certains cas, d'une thoracotomie [4, 16].

Les raisons d’émergence de ce syndrome d'inhalation d'EF au cours des 2 dernières décennies n’étaient pas claires, puisque le voile est porté à travers le monde islamique pour des siècles. Les investigations culturelles ont révélé un changement de la manière de porter le voile et une différence de la technique de sa fixation entre les jeunes filles musulmanes comparées à leurs mères. Les femmes plus âgées utilisent des méthodes de fixation traditionnelles: épingles de sûreté (épingles à nourrice) ou boutons-pression, mais la nouvelle tendance de mode est d'utiliser des épingles droites pointues à tête colorée afin de fixer le foulard ou l’écharpe étroitement en place et les jeunes filles sont, généralement, plus influencées par les nouvelles tendances de mode [11, 17]. En plus, les épingles nouvellement importées sont encore plus fines et plus légères [18]. L'inhalation d'EF est constamment rapportée chez des jeunes adolescentes [2, 13, 19, 20]. Dans notre étude, l'inhalation d'EF a concerné 69 jeunes filles et, de manière surprenante, un jeune garçon de 14 ans. Celui-ci imitait sa sœur aînée en mettant l’épingle dans sa bouche. En 1923, Chevalier Jackson a noté que la cause principale de l'inhalation des épingles était «la négligence de la part des adultes, mettant l’épingle dans la bouche et de la part des enfants, imitant leurs aînés» [12, 21], ceci est toujours valide. Nos patientes étaient âgées en moyenne de 16,5 ans, ce qui rejoint la majorité des études [2, 4, 15, 22, 23]. Les filles de cet âge sont habituellement moins attentives et moins habiles que les femmes adultes, par conséquent, toute perte de concentration au cours de ces manœuvres mène facilement à l'inhalation d’épingle [2, 8, 13]. Ces accidents ne sont pas retrouvés chez les femmes plus âgées, sans doute parce qu'elles deviennent plus habiles et maîtrisent l'ajustement du foulard. Aucune de nos patientes n'a présenté une détresse respiratoire. Les patientes deviennent asymptomatiques après une période de toux intense (syndrome de pénétration) témoignant de la nature non asphyxiante de ce type de CE [4, 8, 14]. Dans la littérature, le taux des patientes asymptomatiques atteint les 10% [8], dans notre étude, il est de 14,5%. La douleur thoracique est rarement rapportée par les patientes (7,2% dans notre étude). Quand elle est présente, elle reflète l'incrustation de l’épingle dans la muqueuse bronchique [19].

Les examens radiologiques de routine doivent inclure une radiographie cervicothoracique et un abdomen sans préparation afin d’écarter la possibilité d'ingestion du CE [13]. Ils permettent facilement le diagnostic de ce type de CE en raison de sa nature métallique et radio-opaque. Les EF sont retrouvées à des niveaux différents. La localisation dans l'arbre bronchique droit est plus logique en raison de sa disposition plus verticale [4, 15], c'est ce que nous avons retrouvé au cours de notre premier travail ayant concerné 16 patientes en 2010 (68,7%) [24]. Pourtant, ce n’était pas le cas ni pour cette étude, où la localisation dans l'arbre bronchique gauche est la plus fréquente (52,9%), ni pour d'autres [4, 7, 12, 23]. Les auteurs attribuent ces résultats au phénomène de Bernouli: la pression négative secondaire à la toux ou le rire est plus importante au niveau de l'arbre bronchique gauche, du fait de son diamètre plus étroit par rapport au droit, ceci crée une pression d'aspiration qui dirigerait l’épingle vers les bronches gauches [7, 12, 19].

Le diagnostic ou la suspicion de CE intra-bronchique impose la pratique d'une endoscopie bronchique en toute urgence, dans un but diagnostique et thérapeutique. Dans le cas d'inhalation d'EF, l'extraction précoce est essentielle puisqu'une extrémité de cet objet est pointue et peut s'incruster dans la muqueuse bronchique lors d'une toux intense ou d'une respiration profonde. Ces épingles risquent aussi de migrer vers les voies aériennes distales et échapper à la visualisation par bronchoscopie [4, 14]. Dans cette étude comme dans d'autres [2, 8, 9, 25], toutes les épingles sont retrouvées tête en bas, bout métallique vers le haut, dans l'arbre bronchique. Ceci est expliqué par la tenue de l’épingle avec extrémité perlée à l'intérieur de la bouche [9, 12, 15]. L'extraction d'un CE pointu est un défi qui requiert une attention particulière. Le succès de l'extraction dépend de l'expérience du bronchoscopiste et de l'anesthésiste [14, 26]. Il n'y a actuellement pas de consensus sur le choix des instruments et des techniques d'intervention pour l'extraction d'un CE trachéobronchique. Cela est fonction de l'expérience des équipes [3, 5]. Dans les cas d'inhalation d'EF, la bronchoscopie rigide (BR) sous anesthésie générale était considérée comme étant la procédure standard d'extraction et la bronchoscopie souple (BS) était rarement utilisée [2, 9, 13, 14, 21]. La BR permet un grand accès aux voies aériennes supraglottiques assurant une oxygénation correcte et un passage facile du fibroscope et des pinces, ce qui permet une extraction rapide et efficace du CE [10, 22, 25]. C'est pourquoi, elle est préférée chez les enfants [22]. Récemment, la BS est devenue la méthode préférée d'extraction des CE trachéobronchiques chez les adultes avec un taux de succès élevé [10, 27–30]. En effet, les nouveaux spécialistes en pneumologie n'ont pas d'expérience avec la BR [10]. Celle-ci est plutôt limitée aux centres disposant d'une unité de chirurgie thoracique [31]. La BS permettra d’éviter l'anesthésie générale, de mieux explorer les voies aériennes distales et d’écourter la durée d'hospitalisation [10, 27]. Il y a peu d’écrits concernant l'apport de la BS sous sédation consciente et anesthésie locale en matière d'extraction des EF inhalées. Dans l’étude rétrospective de Gokirmak et al. [32], le taux de succès de la BS dans l'extraction des EF inhalées est de 73%, dans l’étude prospective d'Al-Ali et al. [27], il est de 56%, alors que dans l’étude d'Al Azzawi [29], il est plutôt de 95%. Dans notre étude, il est de 83,6%.

Une complication potentielle avec la BS sous sédation consciente et anesthésie locale est le risque d'endommager le larynx ou l'arbre trachéobronchique au cours de l'extraction des épingles inhalées [27, 29]. Dans notre étude, nous n'avons pas rencontré de tel problème. Une autre complication possible est le risque de perdre l’épingle au niveau de la gorge et donc l’épingle sera rapidement ingérée [10, 27, 29]. Cette complication est arrivée dans 2 cas dans notre étude. Ceci est expliqué par la faible capacité de préhension des outils da la BS, la grosseur de l’épingle et le fait que la patiente est consciente et donc capable de tousser et d'avaler. La clé d'extraction de l’épingle est de la saisir adéquatement ou de l'enfermer avec la pince ou un panier. Une fois l’épingle saisie, tous les trois (bronchoscope, pince d'extraction et épingle) doivent être retirés simultanément [10, 33]. Un éventail d'accessoires pour BS est actuellement disponible destiné à l'extraction des CE intrabronchiques incluant: des pinces en « dents de rat », pinces « crocodile », paniers à fils métalliques (wire baskets), sondes magnétiques ou cryo-sondes [31].

Dans l’étude d'Al-Ali et al. [27], les auteurs n'ont pas trouvé de différence entre l'utilisation des pinces à biopsie ou des pinces en « dents de rat » pour l'extraction de l’épingle. Le panier n’était pas également l'outil préféré pour l'extraction de l’épingle. Cependant ces outils doivent être disponibles au cours de la manœuvre d'extraction. Dans notre étude, toutes les épingles inhalées ont été extraites soit par des pinces à biopsie, soit par des pinces « crocodile ». Le succès de l'extraction de l’épingle par BS dépendra largement de l'expérience et de la compétence de l'opérateur plutôt que de l'instrument lui-même [10]. Dans notre étude, la manœuvre d'extraction de l’épingle était conférée à nos seniors dans tous les cas. Elle était réussie en première intention dans 73,8% des cas. Elle était laborieuse dans 9,8% des cas nécessitant de refaire la bronchoscopie, souvent du fait d'une mauvaise préparation ou prémédication de la patiente. Un groupe égyptien a proposé une nouvelle méthode d'extraction des EF inhalées par BR sans l'aide de pince: technique de Sersar (technique Mansoura) introduite en 2004 [8, 31, 33, 34]. Elle consiste à incliner la tête de la patiente vers le bas et d'avancer le bronchoscope rigide jusqu’à l'extrémité de l’épingle de manière à ce qu'elle tombe dans la lumière du scope. L’épingle peut être extraite en appliquant un drainage postural (Trendelenburg) et une aspiration. Cependant cette technique ne peut réussir si l'extrémité pointue de l’épingle est incrustée dans la muqueuse bronchique ou si l’épingle est retrouvée dans les bronches distales. Un tiers des EF dans notre série était incrusté dans la muqueuse bronchique. Nous devions d'abord les libérer par une pince avant de les extraire. Le tissu de granulation développé après inhalation de l’épingle à foulard a compliqué 30% des cas dans notre série, mais nous n'avons pas détecté de corrélation entre le délai écoulé après inhalation et l'importance des granulomes. Ces granulomes ne saignent pas facilement en cas de biopsie différemment du cancer [10], donc le saignement ne constitue pas un problème majeur durant l'extraction du CE avec la pince même en présence de granulomes [10].

Le recours à la BR sera nécessaire à chaque fois que l'extrémité pointue de l’épingle est profondément incrustée dans la muqueuse. Ceci ne peut être prévisible avant la réalisation de la BS. Nous avons eu recours à la BR après échec de la BS chez 3 patientes. Le taux de thoracotomie rapporté dans les séries varie entre 1,6 et 18% [2, 9, 13, 32]. Dans la série d'Al-Ali et al. [27], il est de 6%. Dans notre étude, il est de 4,9%. Dans la série de Zaghba et al. [15], il est de 7,69%. Le recours à la thoracotomie n'est pas nécessairement relié à l'utilisation de la BS comme moyen d'extraction des EF inhalées, mais peut être relié à la localisation distale de l’épingle, le retard de consultation après inhalation et la formation de granulomes autour de l’épingle inhalée [27].

## Conclusion

L'utilisation des EF est une pratique culturelle qui comporte un risque sanitaire sérieux chez les jeunes adolescentes qui portent le voile islamique. L'inhalation d'EF est une urgence pneumologique de plus en plus fréquente dans notre contexte marocain. Les cliniciens doivent être avertis de cette forme distincte de CE intra-bronchiques, son diagnostic et les techniques de son extraction. La bronchoscopie souple, en première intention, est une méthode d'extraction de choix, dont le taux de succès peut atteindre les 100% entre des mains expérimentées. Les mesures préventives restent le meilleur traitement pour éviter cet incident dont les conséquences peuvent être dramatiques. Ce sujet nécessite une éducation sanitaire dans les écoles et les médias afin d’éclairer le public quant aux complications liées à cet accident et de sensibiliser les jeunes filles afin de manipuler d’éventuels objets aiguisés hors la bouche. Parmi les solutions qu'on peut proposer: accrocher l’épingle sur les vêtements au lieu de la mettre dans la bouche, la déposer sur une pelote à épingles ou un support magnétique, encourager les jeunes filles à mettre un voile simple ne nécessitant pas la fixation par une épingle métallique. D'autres alternatives existent pour fixer le voile: l'utilisation des broches, des bandes adhésives ou des boutons-pression.

## References

[1] Caidi M, Kabiri H, Lazrek I, El Maslout A, Ben Osman A (2002). Chirurgie des corps étrangers intrabronchiques. Ann Chir..

[2] Kaptanoglu M, Dogan K, Onen A, Kunt N (1999). Turban pin aspiration; a potential risk for young islamic girls. International Journal of Pediatric Otorhinolaryngology..

[3] Pham Van L (2011). Extraction de corps étranger intra-bronchique chez l'adulte par fibroscopie bronchique. J fran Viet Pneu..

[4] El Ftouh M, Souhi H, Achachi L, Jniene A, El Fassy Fihri MT (2010). Inhalation d’épingle: particularités de ce corps étranger. Maroc Médical..

[5] Benjelloun H, Zaghba N, Bakhatar A, Yassine N, Bahlaoui A (2014). Les corps étrangers trachéobronchiques chez l'adulte. Pan African Medical Journal..

[6] Kendja F, Ouede R, Ehounoud H, Demine B, Yapo P, Tanauh Y (2013). Poumons détruits de l'enfant sur corps étrangers: indications et résultats. Chirurgie thoracique et cardio-vasculaire..

[7] Rizk N, Gwely EN, Biron LV, Hamza U (2014). Metallic hairpin inhalation: a healthcare problem facing young Muslim females. Journal of Otolaryngology-Head and Neck Surgery..

[8] Kaptanoglu M, Nadir A, Dogan K, Sahin E (2007). The heterodox nature of “Turban Pins” in foreign body aspiration; the central Anatolian experience. International Journal of Pediatric Otorhinolaryngology..

[9] Uçcan ES, Tahaoglu K, Mogolkoc N, Dereli S, Basozdemir N, Basok O, Turktas H, Akkoclu A, Ates M (1996). Turban pin aspiration syndrome: a new form of foreign body aspiration. Respiratory Medicine..

[10] Üskül TB, Türker H, Arslan S, Selvi A, Kant A (2007). Use of fiberoptic bronchoscopy in endobronchial foreign body removal in adults. Turkish Respiratory Journal..

[11] Ilan O, Eliashar R, Hirshoren N, Hamdan K, Gross M (2012). Turbin pin aspiration: new fashion, new syndrome. The laryngoscope..

[12] Hamad AMM, Elmistekawy EM, Ragab SM (2010). Headscarf pin, a sharp foreign body aspiration with particular clinical characteristics. Eur Arch Otorhinolaryngol..

[13] Murthy PSN, Ingle VS, Edicula G, Ramakrishma S, Shah FA (2001). Sharp foreign bodies in the tracheobronchial tree. American Journal of Otolaryngology..

[14] Soysal O, Kuzucu A, Ulutas H (2006). Tracheobronchial foreign body aspiration: a continuing challenge. Otolaryngology-Head and Neck Surgery..

[15] Zaghba N, Benjelloun H, Bakhatar A, Yassine N, Bahlaoui A (2013). Epingle à foulard: un corps étranger intra-bronchique qui n'est plus habituel. Revue de Pneumologie Clinique..

[16] Arsalane A, Zidane A, Atoini F, Traibi A, Kabiri EH (2009). Deux cas d'extraction chirurgicale de corps étrangers après inhalation d’épingle de foulard. Revue de pneumologie Clinique..

[17] Cobanoglu U, Can M, Melek M (2010). Turban pin aspirations in children in eastern Anatolia. Ind J Thorac Cardiovasc Surg..

[18] El Mustafa OM, Osman WN (2009). A clinical experience with sharp bronchial foreign bodies in Sudanese patients. Sudanese journal of public health..

[19] Ragab A, Ebied OM, Zalat S (2007). Scarf pins sharp metallic tracheobronchial foreign bodies: presentation and management. International Journal of Pediatric Otorhinolaryngology..

[20] Wani ML, Ganie FA, Wani NUD, Ahangar AG, Lone GN, Lone H, Dar AM, Bhat MA, Singh S, Nazeer NU, Wani SN (2013). The pattern, presentation and management of pardah pin inhalation: report from a single center in northern India. Bull Emerg Trauma..

[21] Ludemann JP, Riding KH (2007). Choking on pins, needles and a blowdart: aspiration of sharp, metallic foreign bodies secondary to careless behaviour in seven adolescents. International Journal of Pediatric Otorhinolaryngology..

[22] Al-Sarraf N, Jamal-Eddine H, Khaja F, Ayed AK (2009). Headscarf pin tracheobronchial aspiration: a distinct clinical entity. Interactive CardioVascular and Thoracic Surgery..

[23] Benyan AKZ, Al Hassani FAA, Kareem DR (2012). Sharp pin inhalation in trachea-bronchial tree in women wearing hijab. JPMI..

[24] Hebbazi A, Afif H, El Khattabi W, Aichane A, Bouayad Z (2010). L’épingle à foulard: un nouveau corps étranger intra-bronchique. Rev Mal Respir..

[25] Datema FR, Borgstein J (2009). A new method to solve an old problem: extraction of a sharp foreign body from the lateral basal part of the bronchial tree of a child. International Journal of Pediatric Otorhinolaryngology Extra..

[26] Shad R, Aditya A (2012). Broken safety pin in bronchus-Anaesthesic considerations. Indian J Anaesth V..

[27] Al-Ali MAK, Khassawneh B, Alzoubi F (2007). Utility of fiberoptic bronchoscopy for retrieval of aspirated headscarf pins. Respiration..

[28] Gencer M, Ceylan E, Koksal N (2007). Extraction of pins from the airway with flexible bronchoscopy. Respiration..

[29] Al Azzawi AIA (2013). Utility of fiberoptic bronchoscopy for retrieval of aspirated headscarf pins. European Scientific Journal..

[30] Taha AY (2013). The use of fiberoptic bronchoscope to remove aspirated tracheobronchial foreign bodies: our experience. Case Reports in Clinical Medicine..

[31] Tariq SM, Succony L, Bhatia RS (2012). Spontaneous expulsion of a sharp foreign body. J Bronchol Intervent Pulmonol..

[32] Gokirmak M, Hasanoglu HC, Koksal N, Yildirim Z, Hacievliyagil SS, Soysal O (2002). Retrieving aspirated pins by flexible bronchoscopy. J Bronchol..

[33] Sersar SI (2011). The Egyptian technique revisited (Sersar-Mansoura technique): how to remove some inhaled foreign bodies through rigid bronchoscopy without using a forceps. Rev Port Pneumol..

[34] Sersar SI, Rizk WH, Bilal M, El Diasty MM, Eltantawy TA, Abdelhakam BB, Elgamal AMF, Abou Bieh AA (2006). Inhaled foreign bodies: presentation, management and value pf history and plain chest radiography in delayed presentation. Otolaryngology-Head and Neck Surgery..

